# Decoding Osteosarcoma's Lactylation Gene Expression: Insights Into Prognosis, Immune Dynamics, and Treatment

**DOI:** 10.1155/ancp/6517238

**Published:** 2025-02-21

**Authors:** Cheng Peng, Chaoqun You, Shuang Cao, Linfei Cheng, Jiaji Ren, Jiashi Cao, Jing Wang, Tielong Liu

**Affiliations:** ^1^Department of Orthopedics, Changzheng Hospital, Naval Medical University, Shanghai 200003, China; ^2^Department of Orthopedics, Clinical Medical College, Weifang Medical University, Weifang 261053, Shandong, China; ^3^Department of Orthopedics, Shanghai General Hospital, Shanghai Jiao Tong University School of Medicine, Shanghai 200021, China; ^4^School of Medicine, Anhui University of Science and Technology, Huainan 232001, China; ^5^Department of Orthopedics, No. 455 Hospital of the Chinese People's Liberation Army, The Navy Medical University, Shanghai 200052, China

**Keywords:** gene expression, immune system phenomena, osteosarcoma, prognosis, tumor microenvironment

## Abstract

Osteosarcoma (OS), characterized by a complex tumor microenvironment, poses challenges in treatment, metastasis, and therapy resistance. This study examined the impact of lactylation, a posttranslational modification, on gene expression and tumor behavior in OS, particularly its influence on prognosis, immune cell infiltration, and chemotherapy response. Utilizing data from the Gene Expression Omnibus series accession number 21257 (GSE21257) and the Therapeutically Applicable Research to Generate Effective Treatments on Osteosarcoma (TARGET-OS) datasets, the investigation focused on analyzing the expression profiles of 267 lactylation modifier genes, which were selected from a total of 336 lactylation-related genes compiled from various studies in the literature. The methods included unsupervised clustering using “ConsensusClusterPlus” heatmap generation with “pheatmap” pathway analysis from several databases, and immune cell infiltration assessment using the “single-sample Gene Set Enrichment Analysis (ssGSEA)” function. The research revealed 36 significant lactylation-related genes in OS, categorizing them into two clusters with distinct survival and biological characteristics. One cluster demonstrated poor prognosis due to increased tumor cell proliferation and specific immune cell variations, also showcasing genes that enhance tumor growth and metastasis, thus indicating its aggressive nature and adverse outcomes for patients. These insights are crucial for understanding the molecular mechanisms of OS and identifying therapeutic targets. Therefore, the study elucidates the role of lactylation-related genes in the prognosis, pathogenesis, and treatment response of OS, laying the groundwork for further exploration into potential therapeutic targets and the underlying mechanisms within OS.

## 1. Introduction

Osteosarcoma (OS) is a rare and aggressive bone cancer, primarily affecting adolescents and young adults. Its epidemiology shows a bimodal distribution, with the majority of cases occurring in individuals aged 10–25 years [1]. This malignancy typically originates in the long bones, such as the arms and legs, and often presents with pain and swelling [2]. Typically, the treatment approach for OS encompasses a dual strategy: surgical removal of the tumor, coupled with chemotherapy to address potential residual cancer cells [3]. The prognosis of OS patients can vary, but advances in treatment have improved overall survival rates, with approximately 60%–70% of patients achieving long-term remission 01 [4, 5]. Nevertheless, challenges in treatment endure, as OS can be resistant to chemotherapy, and the disease may spread to other parts of the body, making complete eradication challenging [6]. Furthermore, the aggressive nature of this cancer calls for intricate and frequently strenuous treatment plans, which can profoundly affect the patient's quality of life. This underscores the critical need for more precise and effective management strategies in treating OS [7].

Lactylation, a posttranslational modification originating from lactate, has recently been unveiled on lysine residues of human histones [8]. Through functional analysis of this modification, it has been elucidated as an epigenetic marker capable of instigating M1–M2 polarization in macrophages and facilitating the activation of pluripotency gene expression in the course of reprograming embryonic fibroblasts into induced pluripotent stem cells [8–10]. Lactylation is closely associated with the Warburg effect and has garnered significant attention in cancer research. Warburg effect involves cancer cells primarily utilizing glycolysis, producing lactate even in the presence of oxygen, rather than oxidative phosphorylation for energy [11]. Warburg effect promotes carcinogenesis in OS through complex mechanisms, and an increased level of lactate mainly generated in glycolysis has also been observed [12], indicating that lactylation might play a pivotal role in regulating the progress of OS. Recent researches have focused on targeting lactate metabolism as a potential therapeutic strategy. By regulating enzymes involved in lactylation, scientists aimed to disrupt the unique metabolic profile of cancer cells, potentially making them more vulnerable to conventional therapies like chemotherapy or radiation [13, 14]. A study that aimed to investigate the roles of lactate and lactylation in modulating pyruvate kinase M2 (*PKM2*) function in macrophages has documented the lactylation of several sites on *PKM2*, with particular emphasis on *K62* lactylation attributing regulatory functions to this specific modification. The lactylation of *PKM2* serves to augment its enzymatic activity, diminish the tetramer-to-dimer transition, and limit nuclear distribution, regulating the pro-inflammatory function of macrophages [15]. These investigations underscored the importance of understanding lactylation's role in the tumor microenvironment and how modifying lactylation processes can lead to innovative approaches in cancer treatment, including OS.

In the field of OS research, there has been a notable lack of exploration into lactylation-related gene signatures as a tool for prognosis. Addressing this gap, our study introduces a groundbreaking gene signature derived from lactylation-related data. This innovative approach enhances our ability to predict overall survival and treatment responses in OS, adding a new layer of understanding to this complex disease. Through meticulous gathering and analysis of lactylation-related data, we developed a comprehensive gene signature that effectively predicts patient outcomes. Our extensive research included survival analysis, clinical relevance assessment, and immune cell investigation, all centered around these critical genes. This focus significantly advances the body of knowledge in OS research, marking a pivotal step forward in the field.

## 2. Methods

### 2.1. Source of Data and Collection

The RNA-seq data, along with clinical information from the Gene Expression Omnibus series accession number 21257 (GSE21257) dataset was retrieved from the Gene Expression Omnibus (GEO) database [16] and integrated with the Therapeutically Applicable Research to Generate Effective Treatments on Osteosarcoma (TARGET-OS) dataset obtained from the TARGET-OS database [17]. Subsequently, batch effect removal was conducted utilizing the “limma” and “sva” packages. In line with previous studies [8, 18, 19], we initially identified a comprehensive list of 336 lactylation-related genes. Among these, 267 genes that correspond with the expression profiles found in the combined datasets of GSE21257 and TARGET-OS were selected for further analysis and are detailed in Supporting Information 1: Table S1.

### 2.2. Identification of Prognostic Genes

Gene correlations were assessed utilizing the R packages “survival” and “survminer,” and the outcomes were visualized employing “ggplot2.” Univariate Cox analysis, employing a threshold of *p*  < 0.05, was applied to identify lactylation-related genes associated with OS prognosis. Subsequently, survival analysis was conducted, presenting results if *p*  < 0.001.

### 2.3. Lactylation-Based Consensus Clustering Analysis

The R package “ConsensusClusterPlus” was employed for unsupervised clustering based on lactylation genes significantly associated with OS prognosis. Subsequently, a heatmap was generated using the “pheatmap” package. Kyoto Encyclopedia of Genes and Genomes (KEGG), Hallmark, Biocarta, and Wikipathways datasets were individually obtained from the Molecular Signatures Database (MsigDB). Pathway scoring was carried out using the “Gene Set Variation Analysis (GSVA)” package. “GSVA,” a nonparametric and unsupervised method, was commonly utilized to estimate variations in pathway and bioprocess activity within an expression dataset [20]. A *p*  < 0.05, postcorrection for false discovery rate (FDR), is typically regarded as statistically significant, serving as the criterion for gene selection in “GSVA” analysis.

### 2.4. Prognostic Model Development

Univariate Cox regression was employed to assess the impact of differentially expressed genes (DEGs) on prognosis across distinct clusters. DEGs demonstrating a significant effect on survival rate (*p*  < 0.01) were identified. These were subsequently subjected to the least absolute shrinkage and selection operator (LASSO) Cox regression analysis, utilizing the “glmnet” package, to refine the selection of genes.

### 2.5. Immune Profiling Analysis

The immune cell infiltration status and immunity scores for the samples were obtained using the single-sample Gene Set Enrichment Analysis (ssGSEA) algorithm. These scores facilitated the categorization of samples into high- and low-immune groups. The Estimation of Stromal and Immune cells in Malignant Tumors (ESTIMATE) was applied to determine the stromal score and immune score for distinct cluster groups. In addition, due to the lack of OS-specific immunotherapy datasets, we employed the IMvigor210CoreBiologies dataset to analyze potential immune-related outcomes [21]. This dataset, which focuses on urothelial carcinoma patients, was used to model likely response patterns in high-score and low-score OS groups. This analysis offers insights into potential immune response dynamics in OS patients.

### 2.6. Chemotherapeutic Response Prediction

The chemotherapeutic response for each group was forecasted using the extensive Genomics of Drug Sensitivity in Cancer (GDSC) database. The prediction methodology entailed the use of the “pRRophetic” R package, employing ridge regression to estimate IC_50_. The accuracy of predictions was assessed through 10-fold cross-validation based on the GDSC training set. The parameters defaulted to their standard values, except for addressing batch effects with “combat” and “allSoldTumours” and handling repeated gene expression by substituting them with average values.

### 2.7. Statistical Analysis

One-way analysis of variance and Kruskal–Wallis test, both parametric and nonparametric methods, were employed for comparing two or more groups. Spearman correlation analyses were utilized to compute the correlation coefficient. The Kaplan–Meier method was utilized to construct survival curves for subgroups in each dataset, and the log-rank (Mantel–Cox) test determined the statistical significance of differences. Heat maps were generated using the “pheatmap” function. *p*-Values were two-sided, and those less than 0.05 were considered statistically significant.

## 3. Results

### 3.1. Identification of Lactylation-Related Genes in OS

**Figure 1:**
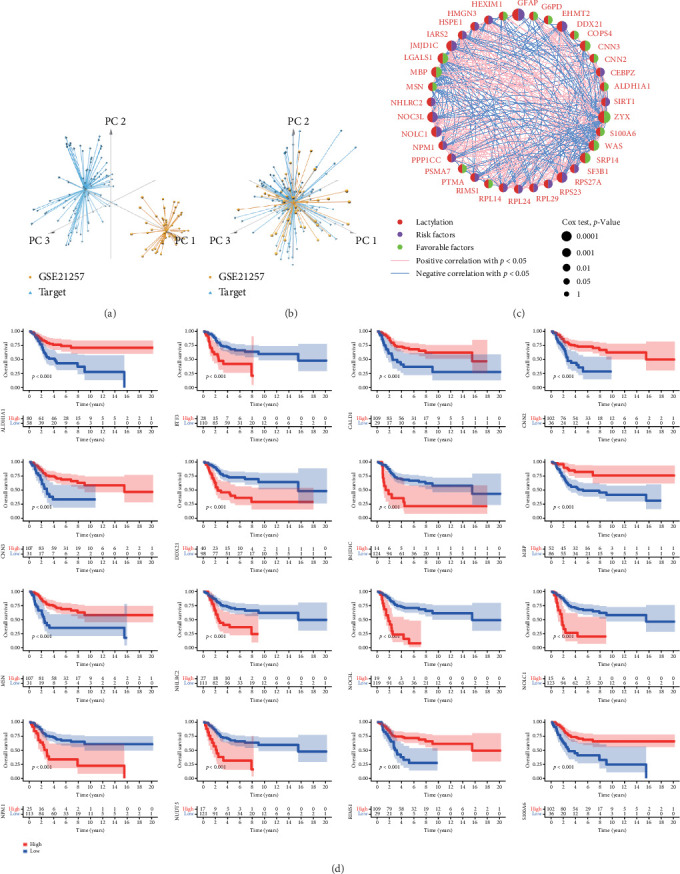
Identification and analysis of lactylation-related genes in osteosarcoma. (A and B) GSE21257 data from GEO were merged with TARGET-OS, and batch effects were removed using R packages “limma” and “sva.” (A) Data before removing batch effects. (B) Data after removing batch effects for data normalization. (C) Correlation analysis between lactylation-modified genes to identify gene expression relationships. (D) Prognosis analysis of lactylation-modified genes, grouped by gene expression levels (*p* < 0.001), to evaluate their impact on patient survival. GEO, Gene Expression Omnibus; GSE21257, Gene Expression Omnibus series accession number 21257; TARGET-OS, Therapeutically Applicable Research to Generate Effective Treatments on Osteosarcoma.

The RNA-seq data and clinical information from the GSE21257 dataset were integrated with TARGET-OS, followed by the removal of batch effects. The TARGET-OS dataset is comprised of 88 samples, encompassing 85 instances with survival analysis and a follow-up duration greater than zero. Similarly, the GSE21257 dataset yielded 53 samples eligible for survival analysis with a follow-up duration exceeding zero. Consequently, a combined dataset consisting of 14,174 genes and 141 samples was generated after integration. The samples from the GSE21257 dataset and TARGET-OS, both before and after batch effect removal, are depicted in Figure 1A,B, respectively. Upon systematic identification, 336 lactylation-related genes were initially pinpointed. Among these, 267 genes that matched the expression profiles within the combined datasets of GSE21257 and TARGET-OS were further analyzed and are cataloged in Supporting Information 1: Table S1. Subsequent correlation analysis and univariate Cox analysis on these genes led to the identification of 36 genes with a significance level of *p*  < 0.05, comprising 16 favorable genes and 20 adverse genes (Figure 1C and Supporting Information 1: Table S1). A prognostic analysis of lactylation-related genes was then conducted, and groupings were established based on gene expression levels. Notably, genes with a significance level of *p*  < 0.001 were presented, delineating eight favorable factors and eight risk factors (Figure 1D).

### 3.2. Relationship Between Gene Expression and Cluster

**Figure 2:**
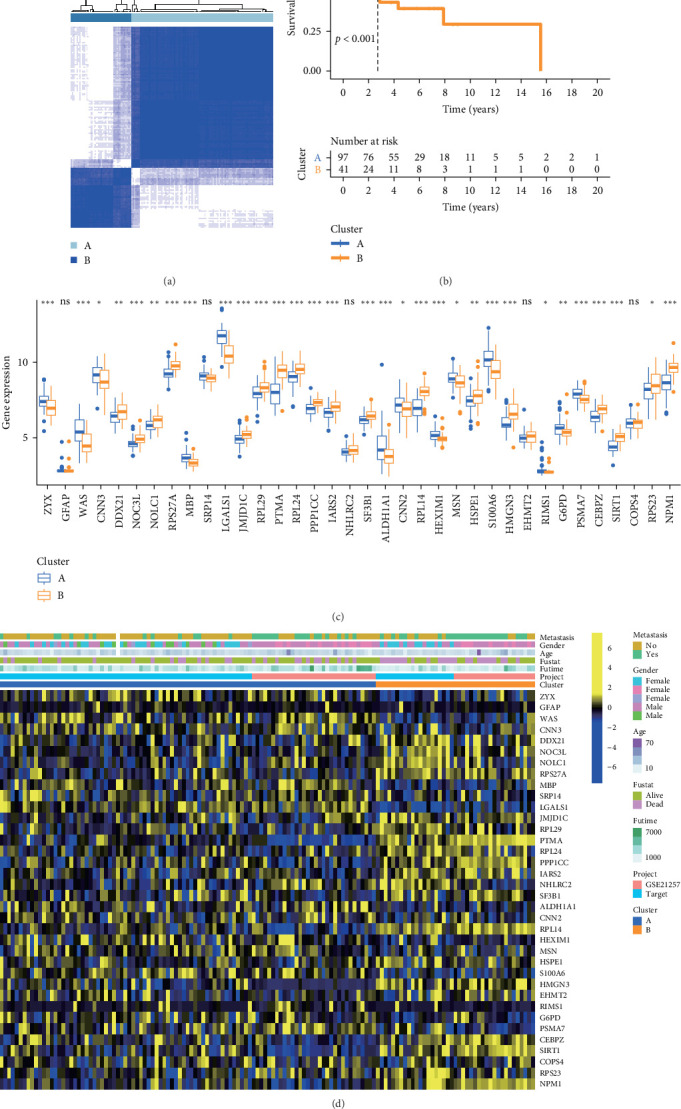
Relationship between gene expression and molecular clustering. (A) Unsupervised cluster typing was performed using the R package “ConsensusClusterPlus,” indicating two distinct clusters. (B) Survival curves for clusters A and B to compare survival outcomes. (C) The expression differences of genes among different clusters were shown (ns, *p*  > 0.05; *⁣*^*∗*^, *p*  < 0.05; *⁣*^*∗∗*^, *p*  < 0.01; *⁣*^*∗∗∗*^, *p*  < 0.001). (D) Heat map showing the relationship between clinical features, gene expression, and clusters.

To identify molecular clusters within OS, we further categorized samples into distinct clusters based on the expression profiles of 36 genes. This analysis revealed the presence of two well-defined clusters (*k* = 2), as illustrated in Figure 2A. Subsequent prognostic analysis highlighted a notable survival advantage in cluster A, contrasting with the considerably poorer prognosis observed in cluster B (Figure 2B). The differential expression of the 36 genes between clusters A and B is depicted in Figure 2C. To further elucidate the clinical significance, we visually portrayed the relationship between gene expression and key clinical features, including metastasis, gender, age, fustat, and futime (Figure 2D). The results showed that many genes, such as *DDX21*, *NOC3L*, *NOLC1*, *RPS27A*, *PTMA*, *RPL24*, and *SIRT1*, were significantly upregulated in cluster B, and the upregulation of these genes was significantly associated with “fustat of dead.”

### 3.3. Pathway Differences Between Different Clusters

**Figure 3:**
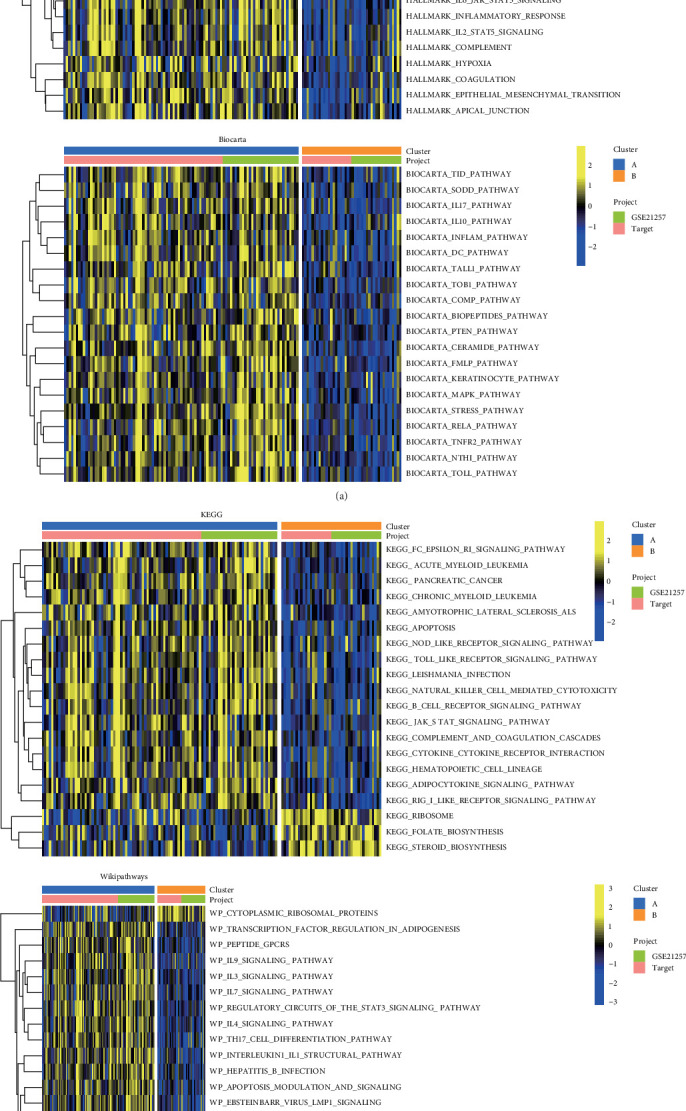
Pathway differences between different clusters. (A) Heat map of pathway contrast between the two clusters based on Hallmark and Biocarta pathway analyses. (B) Heat map of pathway contrast between the two clusters based on KEGG and Wikipathways pathway analyses.

KEGG pathway, Hallmark pathway, Biocarta pathway, and Wikipathways were obtained from the MsigDB database. Pathway scoring was executed utilizing the R package “GSVA.” Comparative analyses between the two clusters revealed distinct pathway differences. The results of the top 20 terms, presented in separate heat maps (Figure 3), showcased notable disparities between the clusters. The findings of the Hallmark pathway demonstrated that cluster A is closely associated with tumor-related pathways, such as Apoptosis, *P53*, and Hypoxia, while cluster B is closely associated with *MYC* targets and *G2M* checkpoint. The Biocarta pathway results showed that cluster A is significantly associated with pathways such as tumor immune dysfunction and exclusion (TID), silencer of death domains (SODDs), *IL17*, *IL10*, and inflammation (INFLAM). The analysis of the KEGG pathway revealed a strong association of cluster A with several pathways, including the FC epsilon RI signaling pathway, acute myeloid leukemia, apoptosis, natural killer cell-mediated cytotoxicity, and B-cell receptor signaling pathway, while cluster B exhibited a significant association with pathways related to the ribosome, folate biosynthesis, and steroid biosynthesis. The results of Wikipathways showed that cluster A is closely relevant to peptide G protein-coupled receptors (GPCRs), Th17 cell differentiation, and apoptosis, while cluster B is closely associated with cytoplasmic ribosomal proteins. The results indicated the close association of clusters with the above typical tumor-associated pathways.

### 3.4. Immune Infiltration Assessment

**Figure 4:**
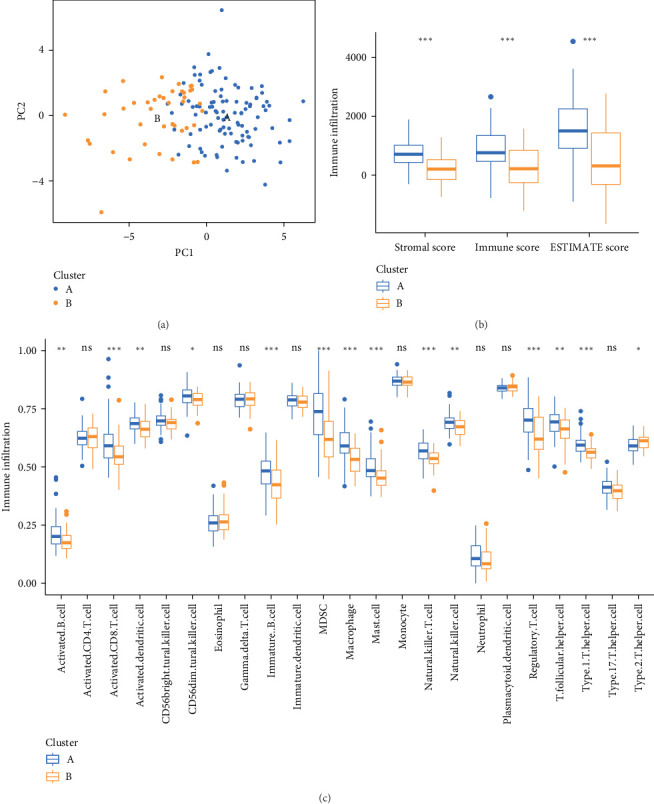
The analysis of immune infiltration. (A) PCA plot showing the distribution of different types of samples. (B) Differences in immune fractions and stromal fractions among types. (C) Differences in the scores of immune cell infiltration among different types (ns, *p*  > 0.05; *⁣*^*∗*^, *p*  < 0.05; *⁣*^*∗∗*^, *p*  < 0.01; *⁣*^*∗∗∗*^, *p*  < 0.001). PCA, principal component analysis.

The principal component analysis (PCA) plot, depicted in Figure 4A, illustrates the distribution of various sample types. To evaluate immune infiltration across different clusters, we assessed the immune score, stromal score, and their cumulative sum. Notably, cluster A exhibited significantly higher average stromal score, immune score, and ESTIMATE score compared to cluster B (Figure 4B), underscoring the meaningful contribution of stromal and immune scores to the classification. We further explored the association between the lactylation-related gene signature and tumor immunity. The results revealed notable disparities in immune cell infiltration between the two clusters, encompassing activated B cells, activated CD8 T cells, immature B cells, myeloid-derived suppressor cells (MDSCs), macrophages, mast cells, natural killer T cells, natural killer cells, and regulatory T cells (Figure 4C).

### 3.5. Functional Analysis for the DEGs

**Figure 5:**
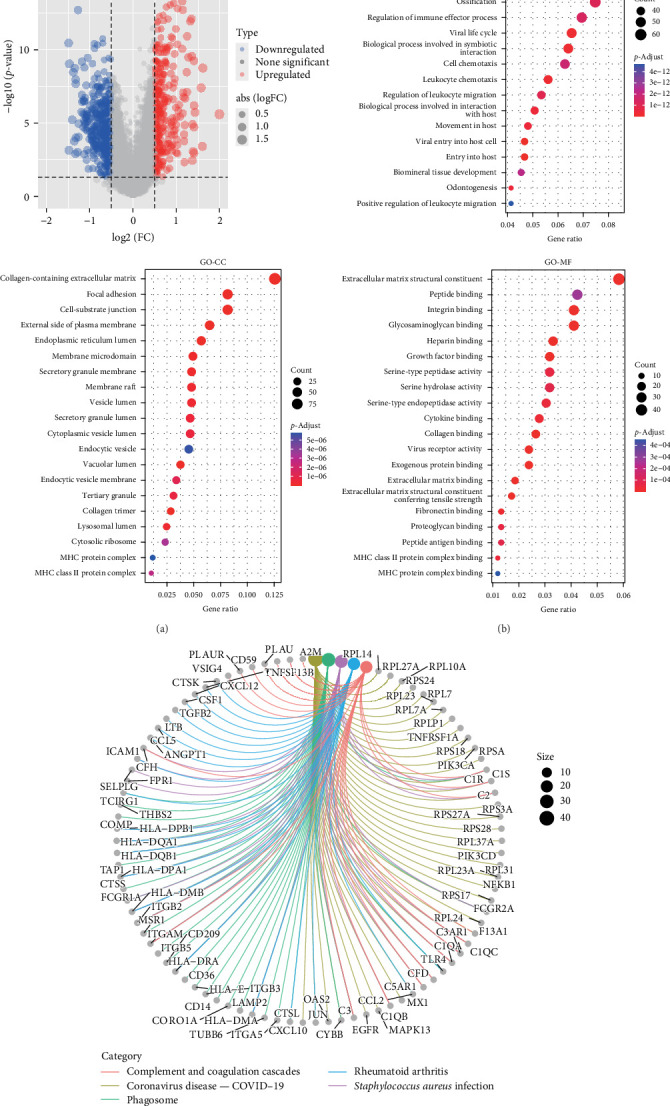
Functional analysis of DEGs. (A) Volcano plot showing upregulated (red) and downregulated genes (blue) to visualize significant gene expression changes. (B) GO enrichment analysis results illustrating the top 20 GO terms in BP, CC, and MF categories to identify associated biological functions. (C) KEGG pathway analysis for the top five pathways to reveal potential biological mechanisms related to the DEGs. BP, biological process; CC, cellular component; DEGs, differentially expressed genes; GO, Gene Ontology; KEGG, Kyoto Encyclopedia of Genes and Genomes; MF, molecular function.

Based on the criteria of a logarithmic fold change (LogFC) greater than 0.5 and a *p*-value less than 0.05, a total of 803 genes were identified as differentially expressed, and volcano plots were constructed, delineating upregulated genes in red and down-regulated genes in blue (Figure 5A). These 803 genes and their related analyses are included in Supporting Information 2: Table S2. Subsequent to this, comprehensive Gene Ontology (GO) and KEGG enrichment analyses were executed to elucidate the pathways and potential mechanisms characterizing the clusters. The top 20 (*p*  < 0.01) GO terms, spanning biological process (BP), cellular component (CC), and molecular function (MF), were visually presented in Figure 5B. The BP results indicated enrichment in “Extracellular Matrix Organization” and “Cell Adhesion,” known to be closely associated with cancer cell invasion and migration [22]. CC terms revealed concentrations in the “Collagen-Containing Extracellular Matrix,” “Focal Adhesion,” “Cell-Substrate Junction,” and the “External Side of the Plasma Membrane.” MF terms, including “Extracellular Matrix Structural Constituent,” “Peptide Binding,” “Integrin Binding,” and “Tensile Strength in Cells,” provided insights into the potential functions of these DEGs in tumor metastasis and signal transduction. Furthermore, the KEGG pathway analysis identified the top five pathways, namely complement and coagulation cascades, coronavirus disease-COVID-19, phagosome, rheumatoid arthritis, and *Staphylococcus aureus* infection (Figure 5C).

### 3.6. Identification of Gene-Clusters Associated With Prognosis and Immune Cell Infiltration in OS

**Figure 6:**
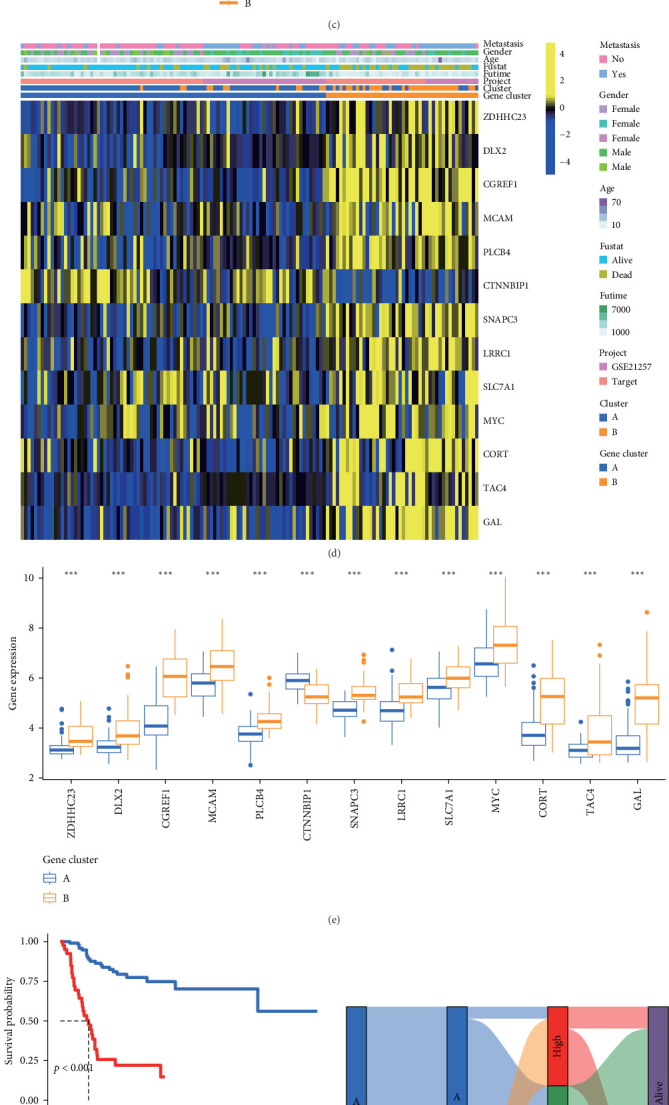
Gene-clusters associated with prognosis and immune cell infiltration in OS. (A) Univariate regression analysis identifying 13 genes associated with prognosis. (B) Clustering results based on the 13 identified genes. (C) Survival analysis showing distinct survival curves between clusters. (D) The heatmap of genes in different clusters. (E) Differential expression of 13 genes between different gene-clusters (ns, *p*  > 0.05; *⁣*^*∗*^, *p*  < 0.05; *⁣*^*∗∗*^, *p*  < 0.01; *⁣*^*∗∗∗*^, *p*  < 0.001). (F) Kaplan–Meier survival curves for high and low score groups. (G) The Sankey diagram of the relationship between subtyping, score, and prognostic status. (H) The results of the correlation between score and immune cell infiltration (red indicates positive correlations; blue indicates negative correlations. The darker the color, the stronger the correlation). OS, osteosarcoma.

In the preceding analysis, the identified DEGs were subjected to univariate regression analysis, resulting in the identification of 13 genes (*p*  < 0.001) for subsequent investigation (Figure 6A and Supporting Information 2: Table S2). Clustering typing results, based on the aforementioned 13 genes, indicated that the optimal value of *K* was 2 (Figure 6B). Prognostic analysis unveiled statistically distinct survival curves (Figure 6C). Notably, patients in cluster A exhibited a survival advantage over those in cluster B, underscoring the protective significance of cluster A in OS patients. Moreover, analysis of gene expression patterns in different clusters, highlighted that, except for *CTNNBIP1* (highly expressed in gene-cluster A), the majority of genes were significantly upregulated in cluster B (Figure 6D). Differential gene expression levels between these clusters were further scrutinized (Figure 6E), confirming the heatmap results. PCA was employed to calculate the score (principal component 1 + principal component 2, PC1 + PC2) based on the aforementioned 13 genes. Subsequent survival analysis showed that higher scores were associated with a worse prognosis (Figure 6F). A Sankey diagram illustrated the relationship between subtyping, score, and prognosis, highlighting that most patients in cluster A, characterized by lower scores, had better outcomes (Figure 6G). Correlation analysis was conducted to explore the relationships between the score and immune cell infiltration (Figure 6H).

### 3.7. The Relationship Between Score and Prognosis

**Figure 7:**
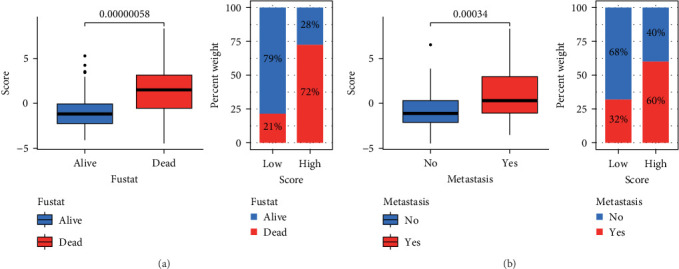
The relationship between score and prognosis. (A) The relationship between score and survival status. (B) Relationship between score and metastasis to illustrate the prognostic relevance of subtype scores.

Figure 7A shows a clear link between the score and patient prognosis, with survivors having notably lower scores than deceased patients. At the last follow-up, 79% of patients with low scores were alive, compared to 72% of those with high scores who had passed away (Figure 7A). Further analysis revealed a significant correlation between higher scores and tumor metastasis, with 60% of patients with high scores experiencing metastasis (Figure 7B).

### 3.8. The Relationship Between Score and Chemokine and Signaling Pathways

**Figure 8:**
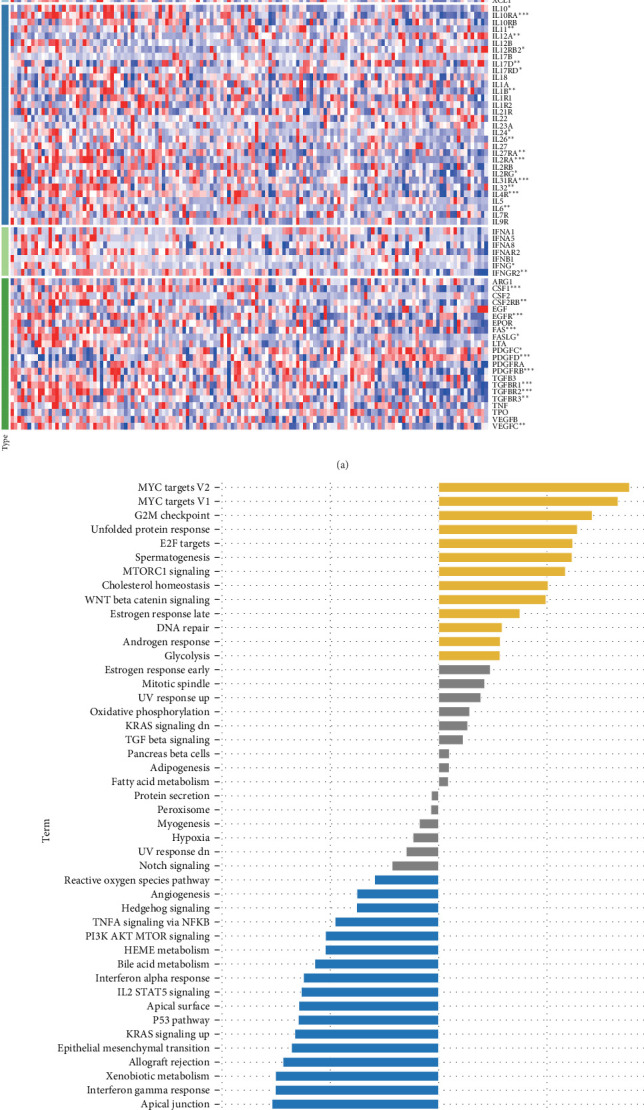
The relationship between score and chemokine and signaling pathways. (A) Expression of chemokines and their receptors in high and low score groups (ns, *p* > 0.05; *⁣*^*∗*^*p* < 0.05; *⁣*^*∗∗*^*p* < 0.01; *⁣*^*∗∗∗*^*p* < 0.001). (B) The correlation of scores with 50 Hallmark channels.

The heatmap shows that low-risk types and low scores correspond to elevated levels of chemokines, interferons, and receptors (Figure 8A). Additionally, Figure 8B presents the “GSVA” analysis, linking scores with 50 Hallmark pathways. Pathways like *MYC* targets V1 and V2, and *G2M* checkpoint positively correlate with the score. This correlation implies increased cellular proliferation and tumor aggressiveness. Specifically, the upregulation of *MYC* targets suggests a higher rate of cancer cell growth and metabolism, while the activation of the *G2M* checkpoint pathway points to accelerated cell cycle progression, potentially leading to rapid tumor development and progression. Conversely, pathways, such as inflammatory response, complement, apoptosis, *IL6 JAK STAT3* signaling, and coagulation, show a negative correlation (Figure 8B).

### 3.9. Analysis of the Immunotherapy Efficacy

**Figure 9:**
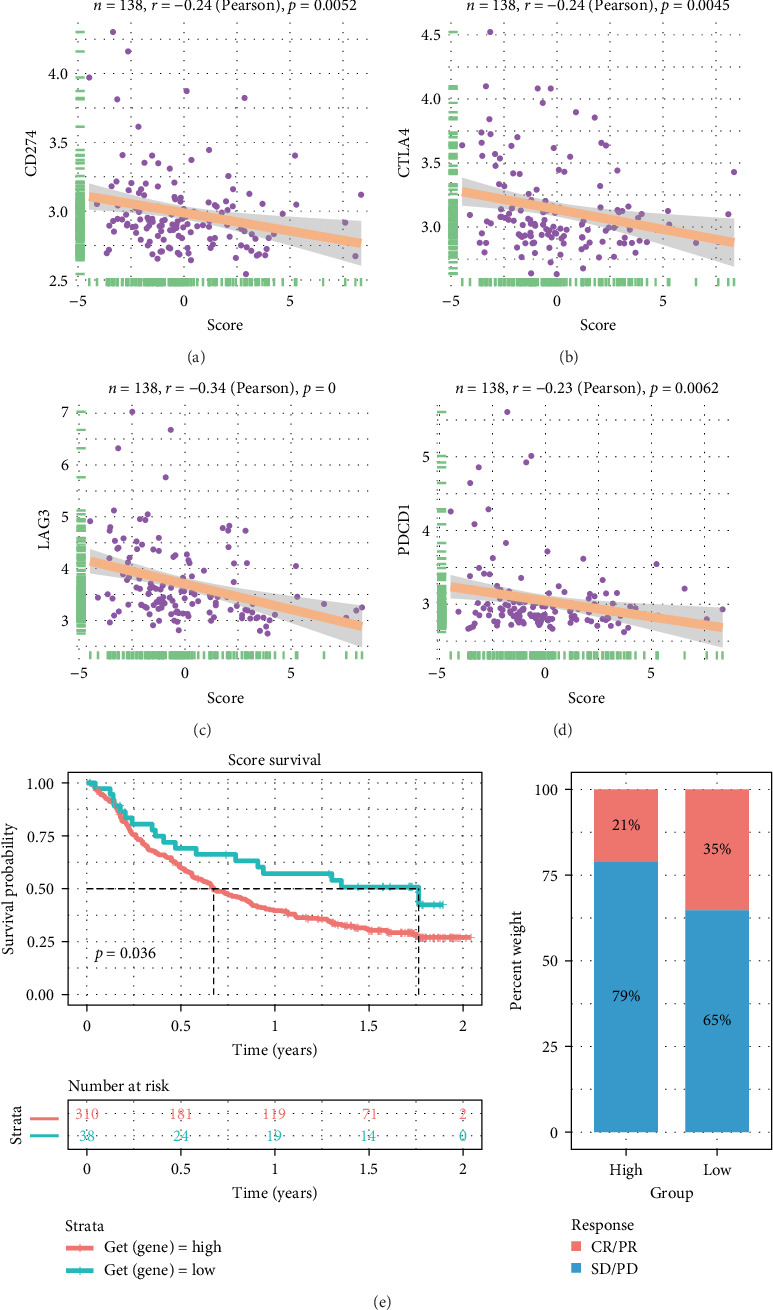
The results of immunotherapy efficacy analysis. (A) Correlation between score and *CD274* expression. (B) Correlation between *CTLA4* expression and score. (C) Correlation between *LAG3* expression and score. (D) Correlation between *PDCD1* expression and score. (E) Comparison of immunotherapy efficacy between high- and low-score groups.

The dot plot illustrates the correlation between the score and the expression of *CD274*, *CTLA4*, and *PDCD1*. It shows that *CD274* (*r* = −0.24, *p*=0.0052), *CTLA4* (*r* = −0.24, *p*=0.0045), and *PDCD1* (*r* = −0.23, *p*=0.0062) are negatively correlated with the score, as depicted in Figure 9A,B,D. However, *LAG3*'s level does not exhibit a significant correlation with the score (Figure 9C). The negative correlation between the score and immune checkpoints, suggesting that patients with higher scores may have lower effectiveness in immunotherapy. Consistent with this result, immunotherapy efficacy analysis, focusing on urothelial cancer immunotherapy showed that patients with higher scores are associated with poorer prognosis (*p*=0.036). This implies that the score could be predictive of patient outcomes in the context of immunotherapy. Additionally, when comparing the high- and low-score groups in terms of immunotherapy efficacy, it is observed that the group with lower scores (35%) has a higher rate of complete or partial response (CR/PR) compared to the high-score group (21%), as illustrated in Figure 9E.

### 3.10. Prediction of Drug Sensitivity for Nonimmunotherapy

**Figure 10:**
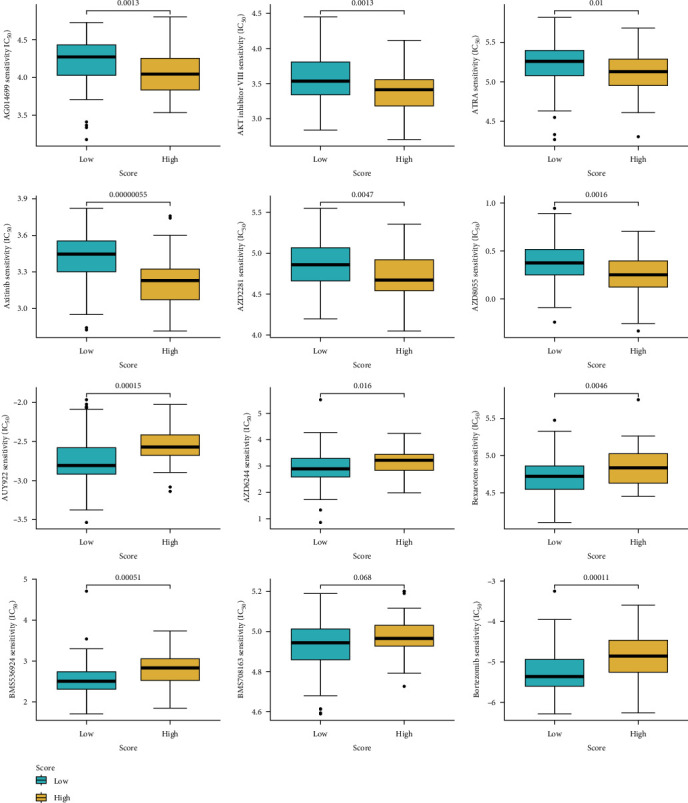
Prediction of drug sensitivity for nonimmunotherapy. The drug sensitivity results of various drugs.

The “pRRophetic” package was employed to predict the IC_50_ values of each sample against various anticancer drugs. The disparities between the IC_50_ values of high- and low-score groups were compared (Figure 10). A higher IC_50_ indicates reduced treatment sensitivity and six plots were generated for each treatment category, illustrating sensitivity and insensitivity. The findings suggested that the low score group may exhibit higher IC_50_ values when treated with AG014699 (*p*=0.0013), *AKT* inhibitor (*p*=0.0013), ATRA (*p*=0.01), Axitinib (*p*=0.00000055), AZD2281 (*p*=0.0047), or AZD8055 (*p*=0.0016) compared to the high-score group. Conversely, the high-score group might demonstrate higher IC50 values when subjected to AUY922 (*p*=0.00015), AZD6244 (*p*=0.016), Bexarotene (*p*=0.0046), BMS536924 (*p*=0.00051), or Bortezomib (*p*=0.00011). Furthermore, the results suggested that there may be no significant difference in sensitivity between the two groups when treated with BMS708163 (*p*=0.068).

## 4. Discussion

This study investigated the lactylation-related genes and the characteristics of these genes in the OS samples. A total of 36 genes were found to be closely associated with the prognosis of OS. Based on the prognostic genes, two subtypes and survival patterns were identified. In addition, the characteristics of immune cell infiltration were assessed. This study comprehensively analyzed the relationship between lactylation-related genes, clinical characteristics, and prognosis in OS.

Lactylation, a novel posttranslational modification of histones and other proteins, has been shown to regulate gene expression and cellular functions in various biological and pathological contexts, such as embryonic development, INFLAM, neuropsychiatric disorders, and cancer [19, 23, 24]. Lactylation is especially relevant for cancer cells, which often exhibit high glycolytic rates and lactate production, known as the Warburg effect [23]. In addition, the epigenetic landscape and the metabolic reprograming of cancer cells can be modulated by lactylation, affecting their proliferation, survival, invasion, and immune evasion [18, 24]. OS is the most common primary bone tumor, characterized by high glycolysis and lactate secretion [25]. A recent study by Wu et al. [25] pinpointed three lactylation-related genes (*SLC7A7*, *MYC*, and *ACSS2*) and their linkage to prognosis and immune infiltration in OS patients. The study highlighted distinct expression levels of these genes in osteoblasts compared to OS cells, hinting at the potential role of lactylation in the tumorigenesis and progression of OS [25]. In our research, we identified a substantial number of genes related to lactylation that exhibit significant correlations with OS, which include risk-associated genes like *SIRT1* and *NPM1*, as well as genes indicative of a favorable prognosis, such as *ZYX* and *G6PD*. The results in this present study demonstrated that detection of the expression level of these genes with biopsy, in clinical practice, might indicate the prognosis and therapy option of patients.

In the current study, the results showed that *SIRT1*, a NAD ± dependent deacetylase that can also delactylate histones and other proteins, is significantly highly expressed in cluster B, indicating that *SIRT1* might be a risk factor for OS patients. Similar to our results, the findings in the previous study demonstrated that *SIRT1* was overexpressed in OS and promotes tumor growth, invasion, and chemoresistance [25]. *SIRT1* also inhibited the antitumor immune response by inhibiting the expression of interferon-stimulated genes [26]. *ZYX* is a cytoskeletal protein that can be lactylated by *EP300*, a histone acetyltransferase that can also catalyze lactylation [27]. *ZYX* lactylation enhances its interaction with *β-catenin* and promotes the epithelial–mesenchymal transition and metastasis of breast cancer cells [27]. A recent study revealed that *ZYX* could inhibit the proliferation, migration, and invasion of OS via inhibiting the *MEK*/*ERK* signaling pathway [28]. Intriguingly, our research further revealed that *ZYX* is predominantly expressed in cluster A, correlating with an increased probability of survival. This suggests that *ZYX* could play a vital role in OS clinical management and emerge as a promising new therapeutic target for OS patients. On the other hand, *G6PD*, a crucial enzyme in the pentose phosphate pathway, is essential for generating NADPH and ribose-5-phosphate, thereby maintaining biosynthesis and redox balance [29]. Our study indicated a significant elevation of *G6PD* in cluster A, which is characterized by a higher survival rate. This aligns with prior research showing *G6PD*'s overexpression in OS and other cancers, contributing to elevated glycolytic activity and lactate production, thereby promoting lactylation [30, 31]. Moreover, *CNN3*, a calcium-binding protein known to influence the cytoskeleton and cell motility, is upregulated in OS [32]. Its overexpression has been linked to poorer prognosis in patients [32]. Our study also identifies *CNN3* as a potential target for lactylation by *EP300*, but the functional consequence of this modification is unclear.

Immune cell infiltration has been demonstrated as a crucial factor in forecasting tumor progression and determining the efficacy of treatment outcomes [33, 34]. Different immune patterns have been identified in OS, such as the high infiltration of macrophages, especially the M0 and M2 subtypes, which are associated with poor prognosis [35, 36]. Other immune cells, such as mast cells, T cells, B cells, and natural killer cells, also play roles in the immune response of OS [35, 36]. Immune-related genes, such as *EGR1*, *CXCL10*, *MYC*, and *CXCR4*, have been found to be potential biomarkers and therapeutic targets for OS [35]. Therefore, understanding the immune infiltration characteristics of OS could help to develop novel and effective immunotherapies for this aggressive disease. The results in this study demonstrated that the activated B cells, activated CD8 T cells, immature B cells, MDSCs, macrophage, mast cells, natural killer T cells, natural killer cells, and regulatory T cells were remarkably relevant to the OS micro-environment, which are consistent with previous studies [35, 36]. Chemokines are a family of small proteins that regulate the migration and activation of immune cells. Chemokine receptors are expressed on the surface of various immune cells and mediate their responses to chemokines. Immune checkpoints are molecules that modulate the immune system by either stimulating or inhibiting the activation of immune cells. Immune checkpoints can be targeted by drugs to enhance antitumor immunity or reduce autoimmune reactions. The research of chemokines, their receptors, and immune checkpoints in OS is aimed at understanding the role of these molecules in the tumor microenvironment, the immune response, and the therapeutic outcome of OS patients. This study demonstrated that many chemokines, their receptors, and immune checkpoints, such as *MYC* targets V2, *MYC* targets V1, and *G2M* checkpoint, were significantly associated with the OS. Our results are parallel with previous studies, demonstrating the involvement of various chemokines and their receptors in the recruitment, polarization, and activity of immune cells in OS. This includes macrophages, T cells, B cells, natural killer cells, and mast cells [37, 38]. Certain chemokines and their corresponding receptors, including *CXCL12*/*CXCR4*, *CCL2*/*CCR2*, and *CCL5*/*CCR5*, have been linked to key aspects of OS progression. These aspects encompass tumor growth, metastasis, angiogenesis, and the development of drug resistance, as documented in studies [37, 39]. Moreover, some immune checkpoints, such as *PD-1*/*PD-L1*, *CTLA-4*, and *TIM-3*, have been found to be expressed in OS and correlated with poor prognosis, immune evasion, and reduced response to immunotherapy [38, 40]. These studies of chemokines, their receptors, and immune checkpoints in OS could provide insights into the molecular mechanisms of OS pathogenesis and immunity. However, a previous study (SARC028) investigating the safety and activity of pembrolizumab, an anti-*PD-1* antibody, in patients with advanced soft tissue and bone sarcomas revealed that the activity of pembrolizumab in bone sarcomas was limited, which might be due to the highly suppressive immune microenvironment. Therefore, the development of a safe and effective strategy for OS is still needed [41].

The findings in this present study showed that the high score group exhibited higher sensitivity when treated by AG014699, *AKT* inhibitor, ATRA, Axitinib, AZD2281, or AZD8055 than the low score group, while the low score group exhibited higher sensitivity when treated using AUY922, AZD6244, Bexarotene, BMS536924, or Bortezomib than the high score group. These drugs have been reported to target different pathways and mechanisms that are involved in OS pathogenesis, such as DNA repair, *PI3K*/*AKT*/*mTOR* signaling, retinoic acid receptor, *VEGF* receptor, *PARP*, *MEK*, *RXR*, *IGF-1R*, and proteasome [42–45]. Some of these drugs have shown promising results in preclinical studies, such as AG014699, which enhanced the cytotoxicity of cisplatin and doxorubicin in OS cells [46]; Axitinib, which inhibited the growth and angiogenesis of OS xenografts [47]; and Bortezomib, which induced cell cycle arrest and apoptosis in OS cells and sensitized them to doxorubicin [48]. Researches of drugs for OS are still ongoing and require further optimization and evaluation of their safety and efficacy.

Although this study comprehensively analyzed the relationship between lactylation-related genes and the prognosis, clinical features, and immune infiltration of OS, there are several limitations in this study. First of all, this study was not validated by in vivo and in vitro experiments, and future studies are needed to verify the results. Second, the limited number of samples used in this study may generate the results of chance. Finally, IMvigor210CoreBiologies, a urothelial carcinoma database, was used to analyze the effect of immunotherapy in this study due to the lack of relevant databases for OS immunotherapy.

## 5. Conclusions

This study pinpointed DEGs in OS related to prognosis, immune infiltration, and immunotherapy, centered around lactylation-related genes. These insights offer a theoretical basis for assessing OS prognosis and further investigating the role of lactylation and related genes in the disease's progression, metastasis, and treatment.

## Data Availability

The datasets supporting the conclusions of this article are available in three repositories. The first dataset is available in the Gene Expression Omnibus (GEO) repository, under series accession number GSE21257, accessible at https://www.ncbi.nlm.nih.gov/geo/query/acc.cgi?acc=GSE21257. The second dataset is part of the Therapeutically Applicable Research to Generate Effective Treatments for Osteosarcoma (TARGET-OS) project, which can be accessed via the National Cancer Institute at https://www.cancer.gov/ccg/research/genome-sequencing/target/studied-cancers/osteosarcoma. The third dataset, IMvigor210CoreBiologies, provides clinical and transcriptomic data on urothelial carcinoma patients treated with the *PD-L1* inhibitor Atezolizumab and is available at http://research-pub.gene.com/IMvigor210CoreBiologies. These datasets were pivotal in our analysis of lactylation-related gene expression and immune profiling in osteosarcoma. Detailed information and specific data subsets used in this study are included within the article and its supporting files.

## Nomenclature

OS: Osteosarcoma
GSE21257: Gene Expression Omnibus series accession number 21257
TARGET-OS: Therapeutically Applicable Research to Generate Effective Treatments on Osteosarcoma
ssGSEA: Single-sample Gene Set Enrichment Analysis
PKM2: Pyruvate kinase M2
GEO: Gene Expression Omnibus
KEGG: Kyoto Encyclopedia of Genes and Genomes
MsigDB: Molecular Signatures Database
GSVA: Gene Set Variation Analysis
FDR: False discovery rate
DEGs: Differentially expressed genes
LASSO: Least absolute shrinkage and selection operator
ESTIMATE: Estimation of Stromal and Immune Cells in Malignant Tumors
GDSC: Genomics of Drug Sensitivity in Cancer
TID: Tumor immune dysfunction and exclusion
SODDs: Silencer of death domains
GPCRs: G protein-coupled receptors
PCA: Principal component analysis
MDSCs: Myeloid-derived suppressor cells
LogFC: Logarithmic fold change
GO: Gene Ontology
BP: Biological process
CC: Cellular component
MF: Molecular function
CR/PR: Complete or partial response.

## Supporting Information

Supporting Information 1 Table S1 presents the prognostic analysis results of lactylation-related genes.

Supporting Information 2 Table S2 details the analysis results of differentially expressed genes.
